# Supplementary material for: Targeted Quantification of Carbon Metabolites Identifies Metabolic Progression Markers and an Undiagnosed Case of SDH-Deficient Clear Cell Renal Cell Carcinoma in a German Cohort
Source: Metabolites. 2021 Nov 9;11(11):764. doi: 10.3390/metabo11110764 (PMC8624007; doi:10.3390/metabo11110764)

Supplementary Figure S1

Spearman correlation matrix of measured metabolites from 318 samples with complete measurements of all 13 metabolites. Cis-aconitate (CisAC) and isocitrate (IsoCA) correlate with citrate (CA), malate (MAL) correlates with fumarate (FUM). Cis-aconitate, isocitrate, and malate were not used for further analyses.

Article

Targeted quantification of carbon metabolites identifies metabolic progression markers and an undiagnosed case of SDH-deficient clear cell renal cell carcinoma in a German cohort

Doreen William[1], Kati Erdmann[2,3,4], Jonas Ottemöller[2], Anastasios Mangelis[5], Catleen Conrad[6], Mirko Peitzsch[6], Evelin Schröck[1,7], Graeme Eisenhofer[6,8], Aristeidis Zacharis[2], Susanne Füssel[2,3], Daniela Aust[1,9], Barbara Klink[1,7,10], Susan Richter[6*]

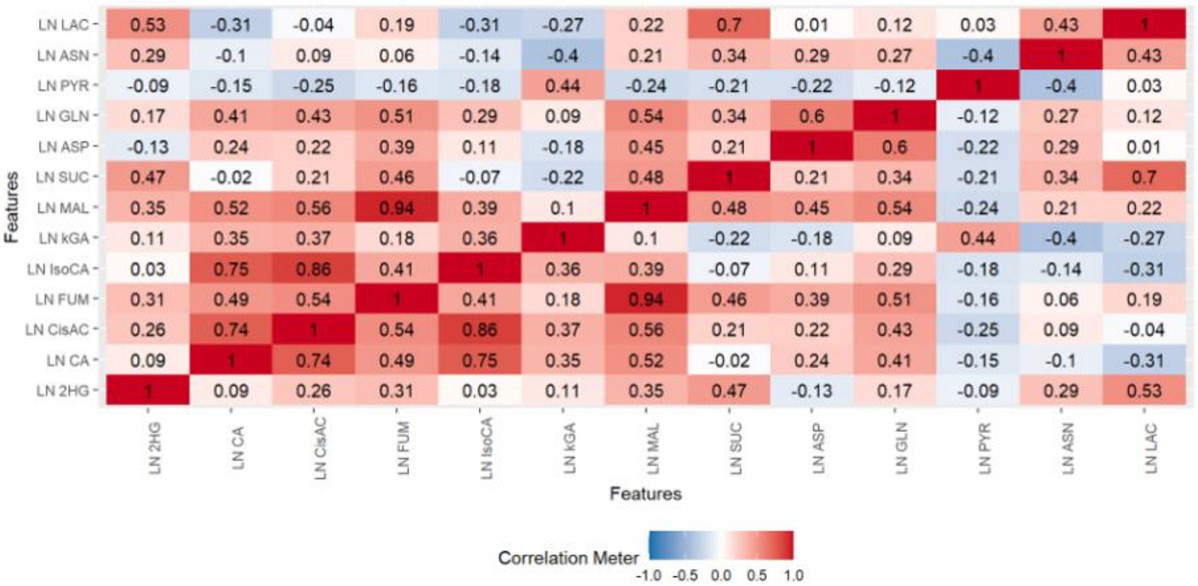

Supplementary Figure S2

Significantly different metabolites between lower (pT1/2) and higher (pT3/4) primary pathological tumour stage across the entire sample set, n = 419, except for pyruvate n = 399 and lactate n = 338. Significance was assessed by Mann-Whitney U test.

Article

Targeted quantification of carbon metabolites identifies metabolic progression markers and an undiagnosed case of SDH-deficient clear cell renal cell carcinoma in a German cohort

Doreen William[1], Kati Erdmann[2,3,4], Jonas Ottemöller[2], Anastasios Mangelis[5], Catleen Conrad[6], Mirko Peitzsch[6], Evelin Schröck[1,7], Graeme Eisenhofer[6,8], Aristeidis Zacharis[2], Susanne Füssel[2,3], Daniela Aust[1,9], Barbara Klink[1,7,10], Susan Richter[6*]

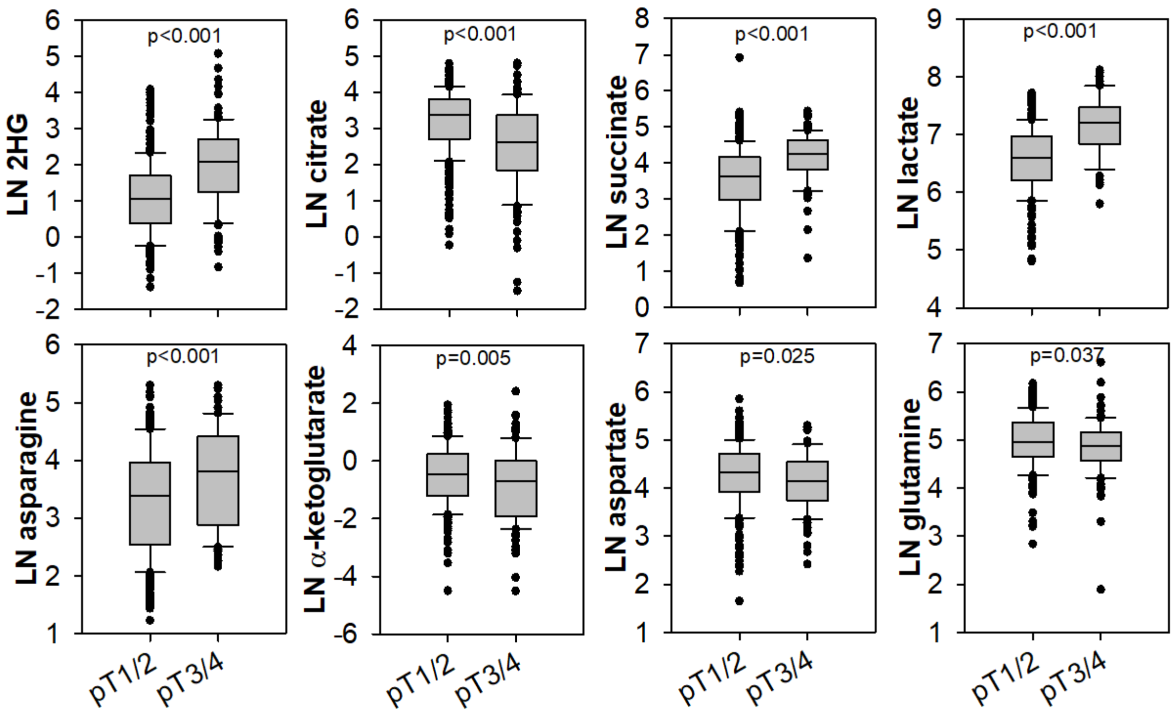

Supplementary Figure S3

Significantly different metabolites between lower (I/II) and higher (III/IV) TNM stage in RCCs across the entire sample set, n = 405, except for pyruvate n = 385 and lactate n = 328. Significance was assessed by Mann-Whitney U test.

Doreen William[1], Kati Erdmann[2,3,4], Jonas Ottemöller[2], Anastasios Mangelis[5], Catleen Conrad[6], Mirko Peitzsch[6], Evelin Schröck[1,7], Graeme Eisenhofer[6,8], Aristeidis Zacharis[2], Susanne Füssel[2,3], Daniela Aust[1,9], Barbara Klink[1,7,10], Susan Richter[6*]

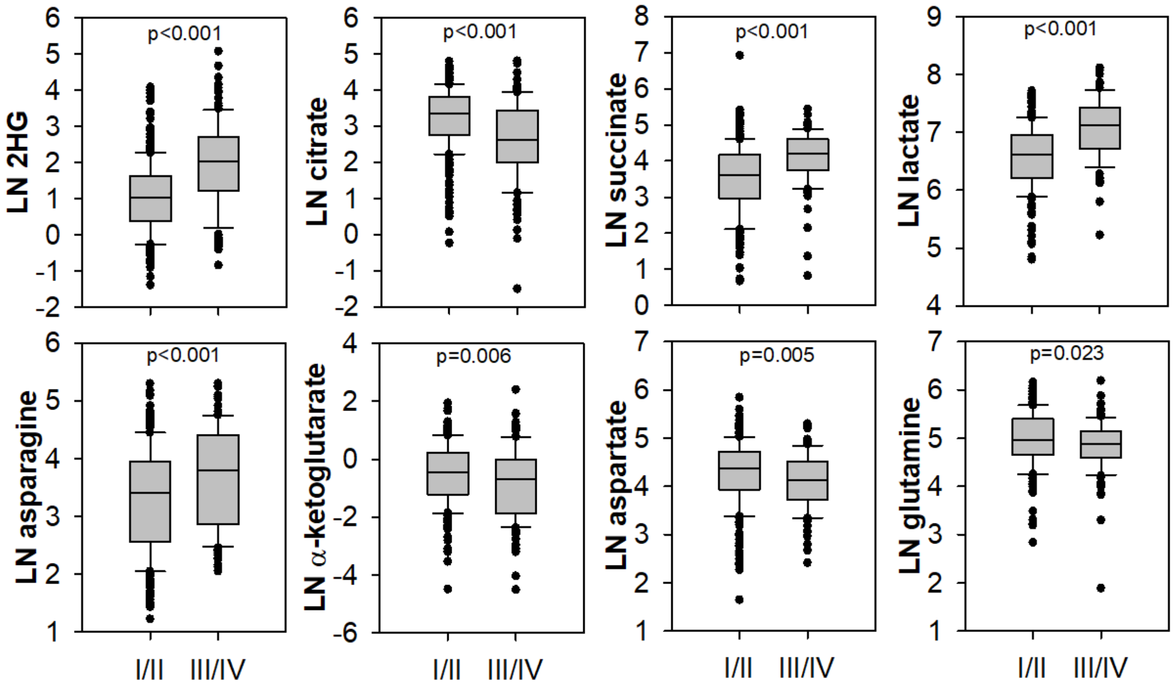

Supplementary Figure S4

Metabolite changes in necrotic (YES) versus non-necrotic (NO) RCC tissue. Significance was assessed by Mann-Whitney U test, n = 419, except for lactate n = 338.

Article

Targeted quantification of carbon metabolites identifies metabolic progression markers and an undiagnosed case of SDH-deficient clear cell renal cell carcinoma in a German cohort

Doreen William[1], Kati Erdmann[2,3,4], Jonas Ottemöller[2], Anastasios Mangelis[5], Catleen Conrad[6], Mirko Peitzsch[6], Evelin Schröck[1,7], Graeme Eisenhofer[6,8], Aristeidis Zacharis[2], Susanne Füssel[2,3], Daniela Aust[1,9], Barbara Klink[1,7,10], Susan Richter[6*]

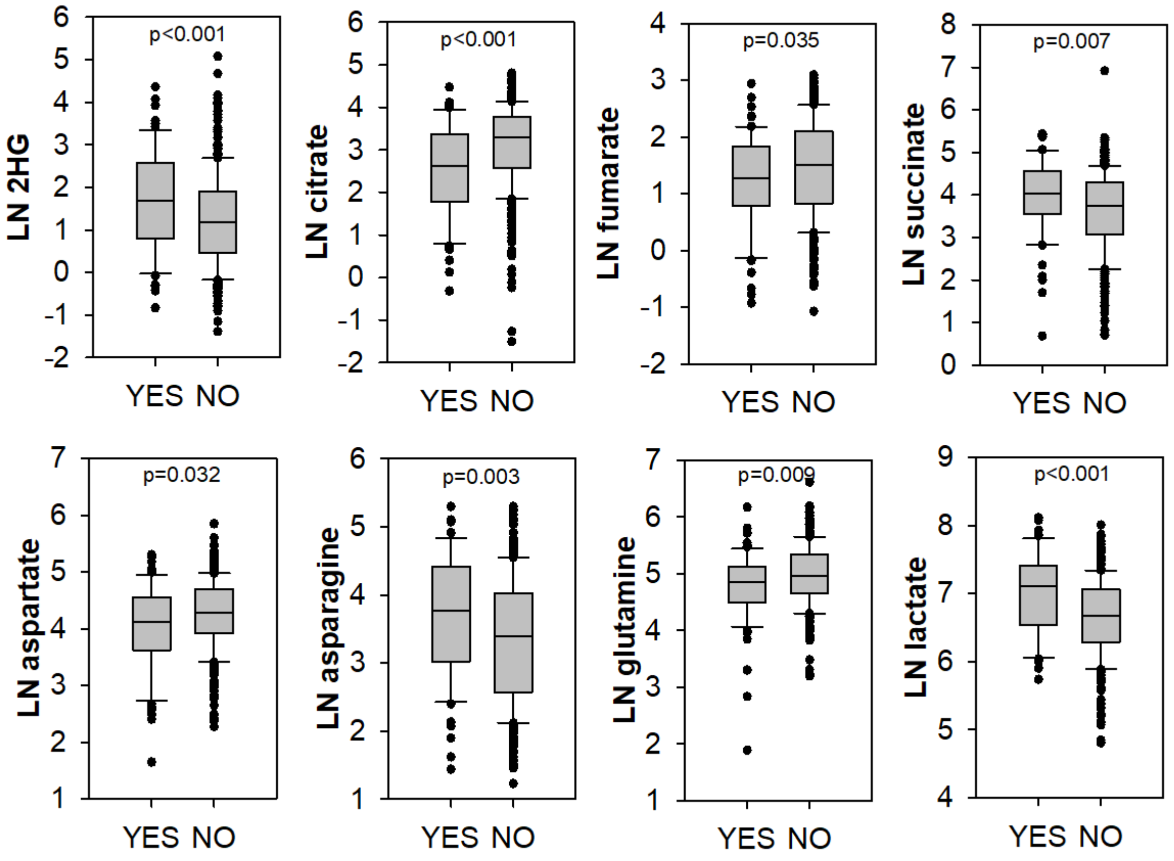

Supplementary Figure S5

Mosaic plot depicting the proportion of primary tumour stages in necrotic and non-necrotic samples. The difference between the necrotic and non-necrotic group is significant by chi-square test, p<0.001.

Article

Targeted quantification of carbon metabolites identifies metabolic progression markers and an undiagnosed case of SDH-deficient clear cell renal cell carcinoma in a German cohort

Doreen William[1], Kati Erdmann[2,3,4], Jonas Ottemöller[2], Anastasios Mangelis[5], Catleen Conrad[6], Mirko Peitzsch[6], Evelin Schröck[1,7], Graeme Eisenhofer[6,8], Aristeidis Zacharis[2], Susanne Füssel[2,3], Daniela Aust[1,9], Barbara Klink[1,7,10], Susan Richter[6*]

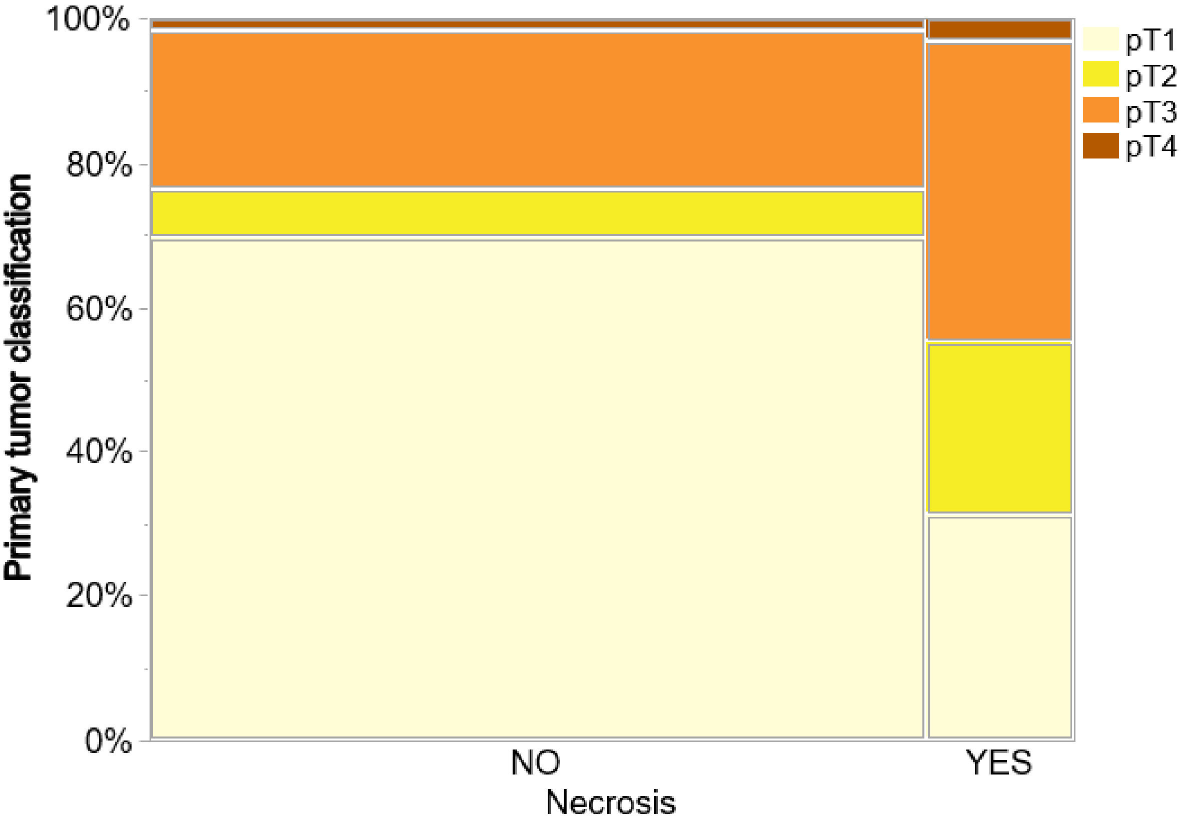

Supplementary Figure S6

Unsupervised hierarchical clustering of metabolites excluding necrotic samples. Colour coding: Necrosis – yes (black), no (grey); Primary tumour stage (pT) and TNM stage – darker is a higher stage; Type – CC (light green), P (dark green), CHR (olive), mixed type (grey).

Article

Targeted quantification of carbon metabolites identifies metabolic progression markers and an undiagnosed case of SDH-deficient clear cell renal cell carcinoma in a German cohort

Doreen William[1], Kati Erdmann[2,3,4], Jonas Ottemöller[2], Anastasios Mangelis[5], Catleen Conrad[6], Mirko Peitzsch[6], Evelin Schröck[1,7], Graeme Eisenhofer[6,8], Aristeidis Zacharis[2], Susanne Füssel[2,3], Daniela Aust[1,9], Barbara Klink[1,7,10], Susan Richter[6*]

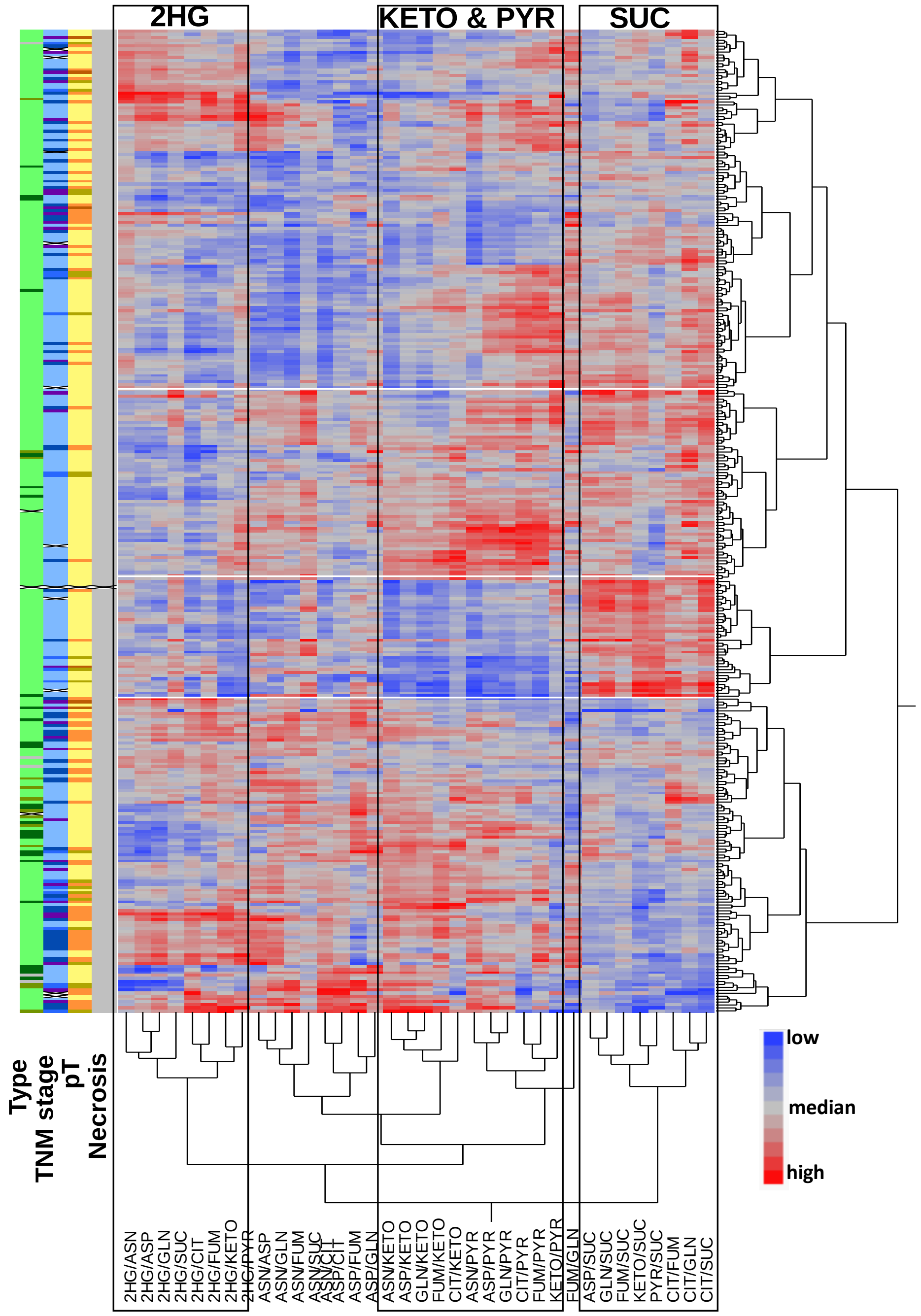

Supplementary Figure S7

Internal validation receiver operating characteristics (ROC) curves for all analytes' classification with the bootstrap forest algorithm between low-TNM-tumours (red lines) and high-TNM-tumours (blue lines). Area under the curve (AUC) score is displayed for all analytes within plots. Training and validation sets were split randomly by 70% and 30% of the original dataset using weighted cross validation.

Article

Targeted quantification of carbon metabolites identifies metabolic progression markers and an undiagnosed case of SDH-deficient clear cell renal cell carcinoma in a German cohort

Doreen William[1], Kati Erdmann[2,3,4], Jonas Ottemöller[2], Anastasios Mangelis[5], Catleen Conrad[6], Mirko Peitzsch[6], Evelin Schröck[1,7], Graeme Eisenhofer[6,8], Aristeidis Zacharis[2], Susanne Füssel[2,3], Daniela Aust[1,9], Barbara Klink[1,7,10], Susan Richter[6*]

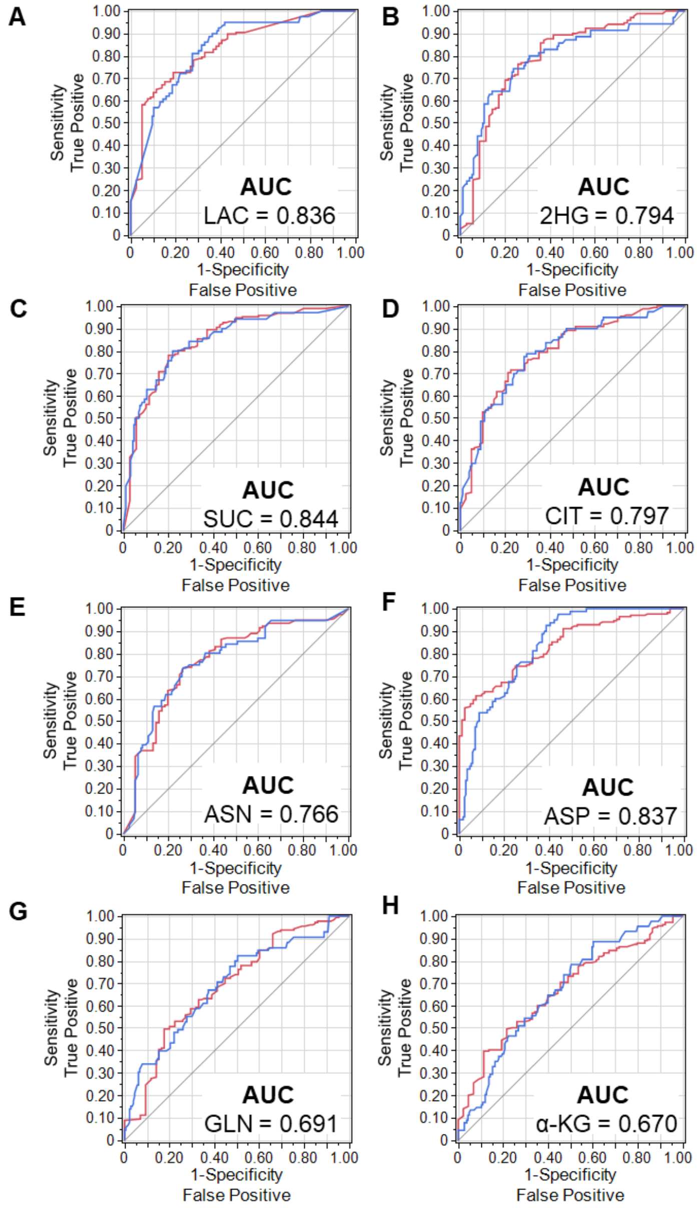

Supplementary Figure S8

Survival analyses for risk assessment based on tumour metabolite levels of lactate (A), succinate (B), 2HG (C), and citrate (D). The figures show probability of disease-specific deaths (which does not include relapse) over time. Metabolite-based predictions were made by the bootstrap forest algorithm, and CSS (n=266) was plotted. Univariable Cox proportional hazards models were used for calculation of hazard ratios (HR) with significance levels at *p<0.05 and **p<0.001.

Article

Targeted quantification of carbon metabolites identifies metabolic progression markers and an undiagnosed case of SDH-deficient clear cell renal cell carcinoma in a German cohort

Doreen William[1], Kati Erdmann[2,3,4], Jonas Ottemöller[2], Anastasios Mangelis[5], Catleen Conrad[6], Mirko Peitzsch[6], Evelin Schröck[1,7], Graeme Eisenhofer[6,8], Aristeidis Zacharis[2], Susanne Füssel[2,3], Daniela Aust[1,9], Barbara Klink[1,7,10], Susan Richter[6*]

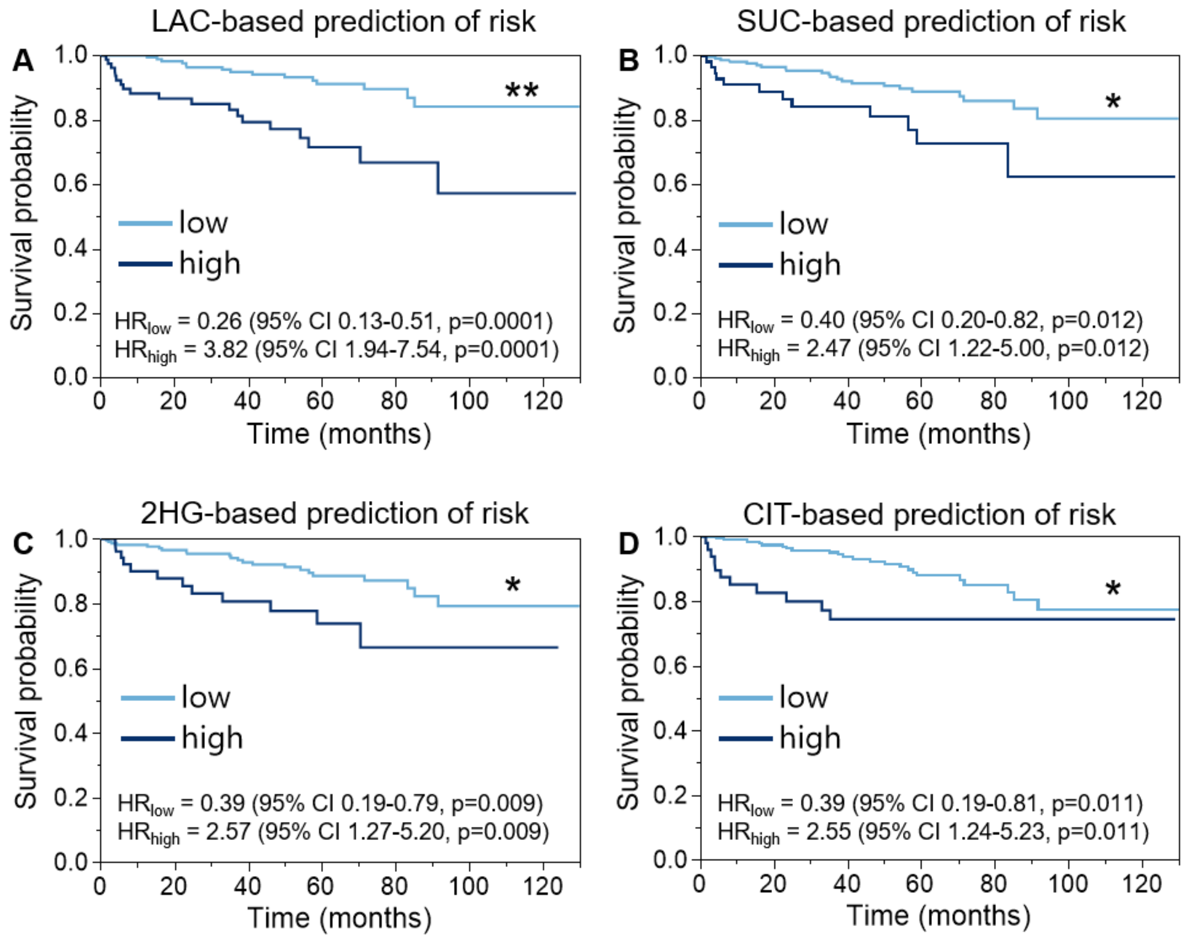

Supplementary Figure S9

ROC curves with areas under the curve (AUC) for the bootstrap forest models predicting risk based on tissue metabolites (n = 405). A′-D′. These predictions for a high or low risk tumour were analysed in respect to CSS (lower panels, n=266); aspartate (ASP, A, A′), asparagine (ASN, B, B′), glutamine (GLN, C, C′), and α-ketoglutarate (α-KG, D, D′). Statistical difference was assessed by log rank test, and significance was considered at *p<0.05. HR – hazard ratios according to Cox proportional hazards model.

Article

Targeted quantification of carbon metabolites identifies metabolic progression markers and an undiagnosed case of SDH-deficient clear cell renal cell carcinoma in a German cohort

Doreen William[1], Kati Erdmann[2,3,4], Jonas Ottemöller[2], Anastasios Mangelis[5], Catleen Conrad[6], Mirko Peitzsch[6], Evelin Schröck[1,7], Graeme Eisenhofer[6,8], Aristeidis Zacharis[2], Susanne Füssel[2,3], Daniela Aust[1,9], Barbara Klink[1,7,10], Susan Richter[6*]

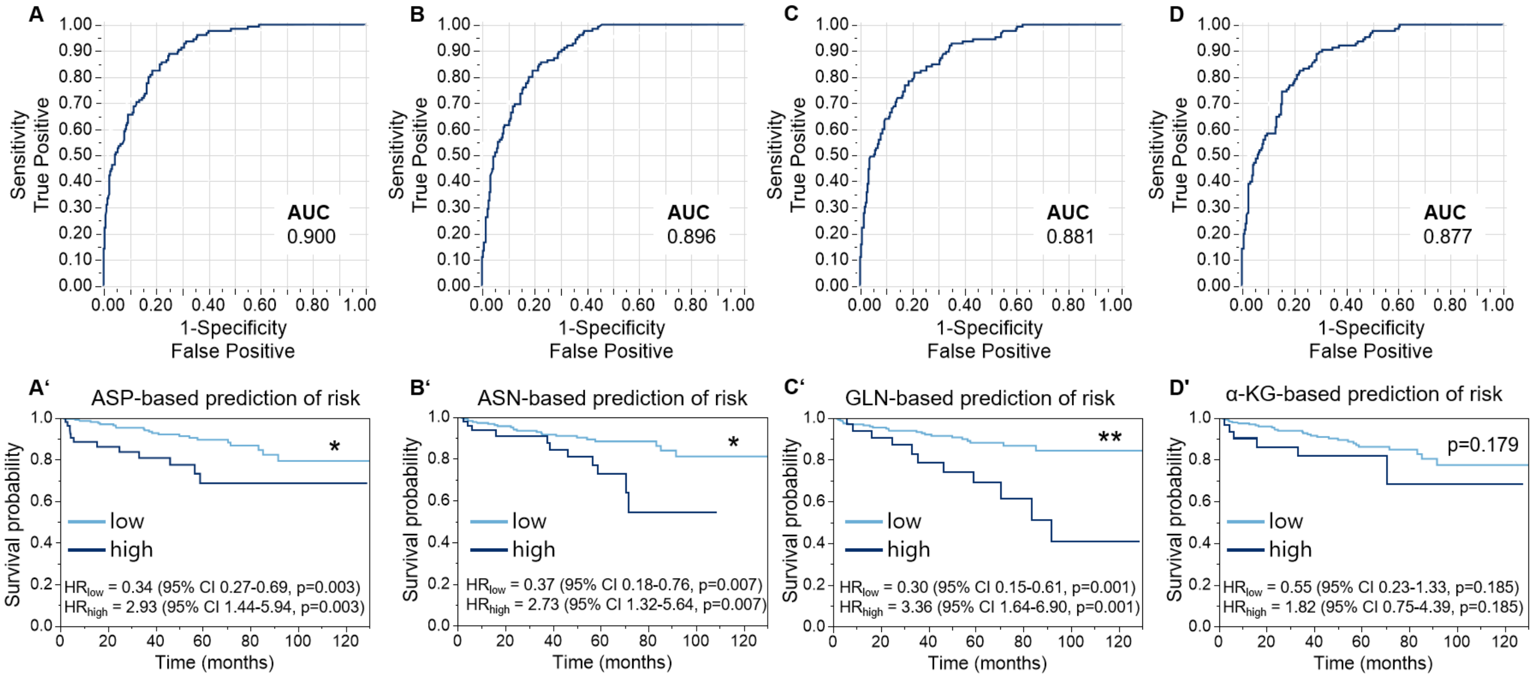

Supplementary Figure S10

Succinate (A) and α-ketoglutarate (B) levels in papRCC type 1 (P1, n=18) versus type 2 (P2, n=15). In five papRCC the type was not differentiated. Statistical significance was assessed by rank sum test.

Article

Targeted quantification of carbon metabolites identifies metabolic progression markers and an undiagnosed case of SDH-deficient clear cell renal cell carcinoma in a German cohort

Doreen William[1], Kati Erdmann[2,3,4], Jonas Ottemöller[2], Anastasios Mangelis[5], Catleen Conrad[6], Mirko Peitzsch[6], Evelin Schröck[1,7], Graeme Eisenhofer[6,8], Aristeidis Zacharis[2], Susanne Füssel[2,3], Daniela Aust[1,9], Barbara Klink[1,7,10], Susan Richter[6*]

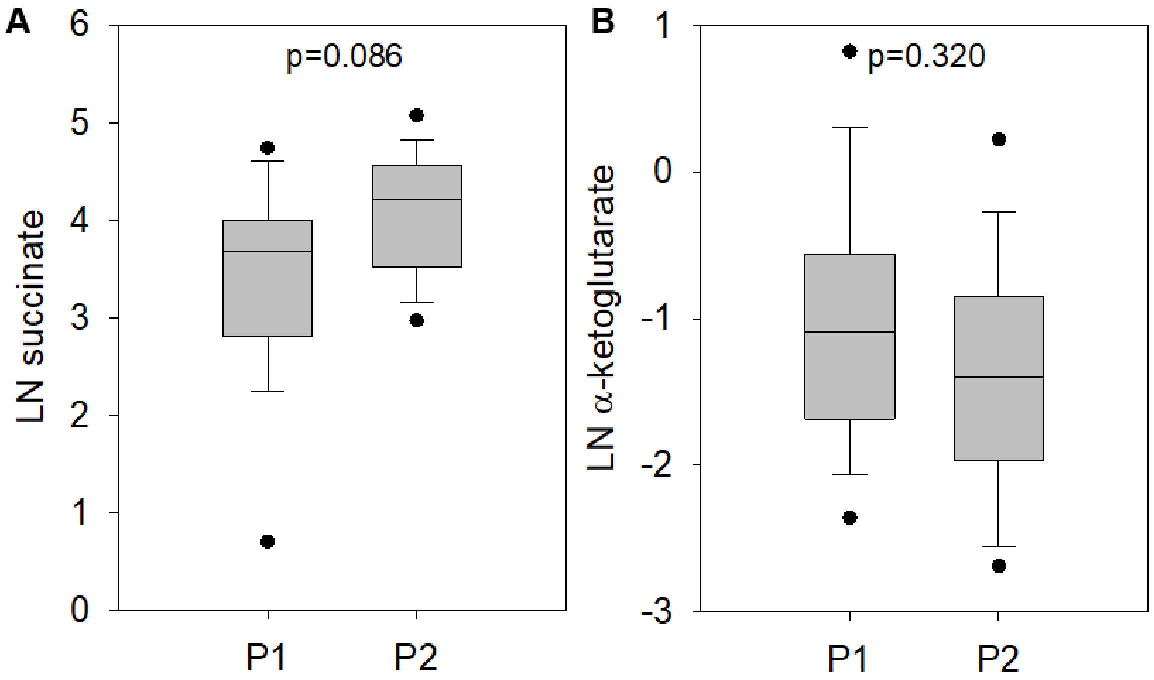

Supplement: Supplementary file 1 [file metabolites-11-00764-s001.zip › metabolites-1391001-supplementary.pdf]
